# Clinical course of sepsis, severe sepsis, and septic shock in a cohort of infected patients from ten Colombian hospitals

**DOI:** 10.1186/1471-2334-13-345

**Published:** 2013-07-24

**Authors:** Alba Luz León, Natalia Andrea Hoyos, Lena Isabel Barrera, Gisela De La Rosa, Rodolfo Dennis, Carmelo Dueñas, Marcela Granados, Dario Londoño, Ferney Alexander Rodríguez, Francisco José Molina, Guillermo Ortiz, Fabián Alberto Jaimes

**Affiliations:** 1 Universidad de Antioquia, Medellín, Colombia; 2 Universidad del Valle y Hospital, Universitario del Valle, Cali, Colombia; 3 Hospital Pablo Tobón Uribe, Medellín, Colombia; 4 Pontificia Universidad Javeriana y Fundación Cardio Infantil, Bogotá, Colombia; 5 Universidad de Cartagena, Hospital de Bocagrande y Clínica Madre Bernarda, Cartagena, Colombia; 6 Fundación Valle de Lili, Cali, Colombia; 7 Pontificia Universidad Javeriana y Hospital Universitario San Ignacio, Botogá, Colombia; 8 Universidad Pontificia Bolivariana y Clínica Universitaria Bolivariana, Medellín, Colombia; 9 Hospital Santa Clara, Bogotá, Colombia; 10 Departamento de Medicina Interna y Grupo Académico de Epidemiología Clínica (GRAEPIC), Universidad de Antioquia y Hospital Pablo Tobón Uribe (Unidad de Investigaciones), Medellín, Colombia

**Keywords:** Severe sepsis, Septic shock, Progression, GEE, Cox regression

## Abstract

**Background:**

Sepsis has several clinical stages, and mortality rates are different for each stage. Our goal was to establish the evolution and the determinants of the progression of clinical stages, from infection to septic shock, over the first week, as well as their relationship to 7-day and 28-day mortality.

**Methods:**

This is a secondary analysis of a multicenter cohort of inpatients hospitalized in general wards or intensive care units (ICUs). The general estimating equations (GEE) model was used to estimate the risk of progression and the determinants of stages of infection over the first week. Cox regression with time-dependent covariates and fixed covariates was used to determine the factors related with 7-day and 28-day mortality, respectively.

**Results:**

In 2681 patients we show that progression to severe sepsis and septic shock increases with intraabdominal and respiratory sources of infection [OR = 1,32; 95%IC = 1,20-1,46 and OR = 1.21, 95%CI = 1,11-1,33 respectively], as well as according to Acute Physiology and Chronic Health Evaluation II (APACHE II) [OR = 1,03; 95%CI = 1,02-1,03] and Sequential Organ Failure Assessment (SOFA) [OR = 1,16; 95%CI = 1,14-1,17] scores. The variables related with first-week mortality were progression to severe sepsis [HR = 2,13; 95%CI = 1,13-4,03] and septic shock [HR = 3,00; 95%CI = 1,50-5.98], respiratory source of infection [HR = 1,76; 95%IC = 1,12-2,77], APACHE II [HR = 1,07; 95% CI = 1,04-1,10] and SOFA [HR = 1,09; 95%IC = 1,04-1,15] scores.

**Conclusions:**

Intraabdominal and respiratory sources of infection, independently of SOFA and APACHE II scores, increase the risk of clinical progression to more severe stages of sepsis; and these factors, together with progression of the infection itself, are the main determinants of 7-day and 28-day mortality.

## Background

Infection is the pathologic phenomenon induced by microorganisms, and sepsis is the systemic response to infection. Severe sepsis occurs when organ dysfunction appears, and septic shock is a sustained reduction of arterial blood pressure that impairs adequate tissue perfusion [1,2]. In previous years, the diagnosis and treatment of sepsis appeared chaotic, partly because of a lack of uniform terminology [1,3]. Consequently, one of the most important advancements was the ACCP/SCCM consensus, which unified the criteria and definitions for diagnosing the syndrome [1]. The process has traditionally been understood as a linear sequence spanning different clinical stages [4], from infection to septic shock. However, with few exceptions such a clinical trial carried out almost two decades ago [5], some theoretical models with mathematical simulations [6,7], and cohort studies with highly selected patients, whether by ICU admission [8] or by the specific diagnosis of pneumonia [9]; a formal and adequate characterization of the potential progression from infection without systemic manifestations to septic shock or death has not been attempted. To understand this clinical behavior, and to know the timeline and the timing of relevant events within the sepsis syndrome, might be a cornerstone of the real dynamic behind infection and host’s response.

In Colombia, we have found some particular features that suggest differences in the natural history and clinical course of bacterial infections with regard to other places in the world [10,11]. Under these circumstances, which are probably valid for most of the third world, complete clinical characterization of that potential progression may make it possible to propose more effective strategies to face the problem. This would be especially valuable for reducing mortality, which varies with the clinical stages: in patients with infection without sepsis, with sepsis, with severe sepsis, and with septic shock, the mortality rates are 3%, 7,3%, 21,9%, and 45,6%, respectively [11]. The aim of this research is to describe the evolution of the stages of sepsis in a multicenter cohort of patients with bacterial infections requiring hospitalization. The study also aims to identify the determinants of the progression of these stages, as well as their relationship with 7 and 28-day mortality.

## Methods

### Settings and study design

This is a secondary analysis of the study *The Epidemiology of Sepsis in Colombia*[11], which is a prospective multicenter cohort study with patients admitted to ten hospitals in the four main cities of Colombia from September 1, 2007, to February 29, 2008. We included patients from emergency rooms, ICUs, and hospital wards covering both community- and hospital-acquired infections. Patients were considered eligible if they were 18 years or older; had a probable or confirmed diagnosis of infection according to medical records; or had changes in temperature (>38 or <36°C) or hypotension without a specific cause. Furthermore, as definitive inclusion criterion, patients must have had an infection that fulfilled standard Centers for Disease Control and Prevention definitions [12]. Patients were excluded if they refused to participate, were screened for eligibility ≥ 24 hrs after suspicion of infection, stayed 48 hrs in another institution immediately before the current hospitalization, were not available for 28-day follow-up, were discharged < 24 hrs after hospitalization, their diagnosis changed toward a noninfectious disease during hospitalization, or were previously recruited in the study. Hospital-acquired infections were defined as those not present or incubating at the time of admission to the hospital, i.e., infections that become evident 48 hrs after admission. The study protocol was approved by the ethical committee of the Medical Research Center (University of Antioquia). Oral informed consent was obtained in all hospitals except in two in which written informed consent was requested.

### Data collection, evaluation, and quality control

There were one or two trained nurses, according to the number of beds, in each hospital. They followed a study protocol standardized twice in 2-day workshops developed within a 3-month pilot study, which was conducted immediately before starting the recruitment. In each hospital, there was also a clinician coinvestigator who was in charge of checking data accuracy and consistency as well as the patient’s diagnosis. In addition, the case report forms were checked and revised weekly in a double-entry form in the Data Coordinating Center (Universidad de Antioquia). Any inconsistency, inaccuracy, or missing data implied returning the specific case report form to the coinvestigator for correction within the next week after the Data Coordinating Center review. There was also on site evaluation during the first month of the study at each hospital by one of the coprincipal investigators. At the recruiting areas of the hospitals, all the inpatients were actively screened for the presence of infection. The severity of illness was assessed using the Acute Physiologic and Chronic Health Evaluation II score [13], and the frequency and magnitude of organ dysfunction was measured with the Sequential Organ Failure Assessment score [14], both determined within the first 24 hrs after enrollment of the patient. We recorded also demographic characteristics, first admission diagnosis, comorbidities and clinical status as infection, sepsis, severe sepsis or septic shock daily during the first seven days (Additional file 1). For this clinical status classification, those laboratory tests not requested were considered as normal. For patients discharged before 28 days, their vital status was confirmed by telephone call or outpatient control.

### Study outcomes

Primary outcomes: clinical progression from infection to sepsis, severe sepsis or septic shock and 7-days mortality. Secondary outcome: 28-days mortality.

### Statistical analysis

Categorical and ordinal variables were expressed as proportions; continuous variables as means with standard deviations or medians with interquartile ranges, according to data distribution. In order to identify the determinants of progression across different clinical stages of sepsis, it is necessary to consider a longitudinal data analysis. Longitudinal data analyses come from studies in which the outcome variable is measured on the same individual at several occasions (e.g., daily clinical stage) and, consequently, observations are not independent of each other. With the method of General Estimating Equations (GEE), the correction for the dependency of observations is done by assuming a correlation structure for the repeated measurements of the outcome variable. These correlation structures vary from an interchangeable (i.e., the correlations between subsequent measurements are assumed to be the same) to an unstructured one (i.e., no particular correlation shape is assumed and all possible correlations between repeated measurements has to be estimated) [15]. Therefore, we estimated the odds of progression of clinical stages with a general estimating equations (GEE) model for a binomial distribution, assuming an interchangeable correlation between measures and a robust (Huber-White) estimator of variance [16,17]. The outcome variable was the daily progression of the clinical condition over the first week (infection without sepsis, sepsis, severe sepsis, septic shock, and death); and we regarded as independent variables those which, as the literature suggests, are potentially related to that progression [8,18,19]: age; sex; place of acquisition of infection; source of infection; comorbidities (at least one of the following: HIV/AIDS, trauma or surgery during the past 30 days, congestive heart failure, organ transplantation, cirrhosis, use of steroids or chemotherapy during the last year, drug addiction or alcoholism, chronic obstructive pulmonary disease, chronic renal failure and/or dialysis, diabetes mellitus, or history of cancer during the past year); SOFA and APACHE II scores; and type of microorganism identified in blood.

As supported by the GEE model, we assumed a constant odds of progression both among stages and within days, and we also considered that the data were missing completely at random. As a sensitivity analysis, the model was fitted with deaths during the first week considered both as missing data and also as a fifth stage, without significant changes in the results. On the other hand, in the literature it is supported that GEE analysis is robust against a wrong choice for a correlation structure. It means that it does not matter which correlation structure is chosen, the results of the longitudinal analysis will be more or less the same [20,21]. However, as a sensitivity analysis, we fitted GEE models also assuming either an unstructured or an “independent” correlation structure, without significant changes in the results.

Cox regression analysis was used to determine the effect of clinical progression from one stage to another (time-dependent covariate) on first-week mortality, adjusted for fixed variables such as age, sex, place of acquisition of infection, source of infection, comorbidities, SOFA, APACHE II, and type of microorganism identified in blood. These results were verified with a Cox model for 28-day mortality with the clinical stages on the first day as fixed variables. Patients discharged alive from hospital were considered censored, and the proportional-hazards assumption was verified by the Schoenfeld residuals test, as well as all potential interactions between variables [22]. All measures of association, OR and HR, were accompanied by their corresponding 95% confidence intervals; all statistical analyses were performed with STATA 12.1 (Stata Corp. College Station, TX, USA).

## Results

Of the 2681 patients enrolled, 136 (5.1%) were classified as having infection without sepsis, 575 (21.4%) with sepsis, 1576 (58.8%) with severe sepsis, and 394 (14.7%) with septic shock at the moment of inclusion in the study. The mean age was 55 yrs (SD = 21 yrs), 1365 (51%) were female, the mean Acute Physiologic and Chronic Health Evaluation II (APACHE II) score was 11.5 (SD = 7), and the mean Sequential Organ Failure Assessment (SOFA) score was 3,8 (SD = 3). The most common associated conditions were trauma or surgery in 28,5% (n = 764), diabetes mellitus in 15,2% (n = 408), chronic renal disease in 10,5% (n = 281), heart failure in 9,9% (n = 266), and chronic obstructive pulmonary disease in 9,9% (n = 265). Among the total cohort, 879 patients (33%) did not have any comorbidity. The overall 7-day and 28-day mortality rate was 9.2% (n = 247) and 18.5% (n = 497), respectively.

**Figure 1:**
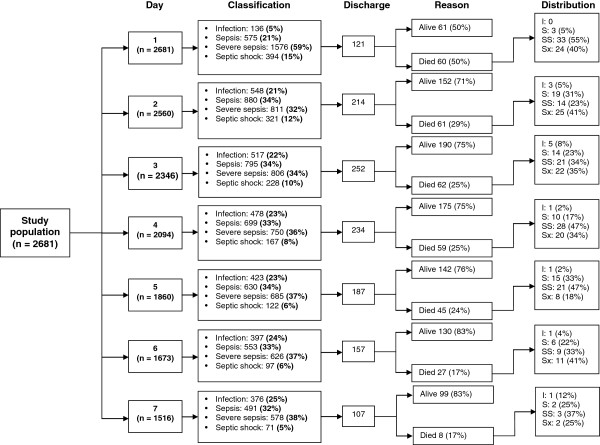
Distribution of patients according to stages of infection (infection without sepsis [I], sepsis [S], severe sepsis [SS], and septic shock [Sx]) for each day of follow-up.

Considering the clinical stage at the moment of inclusion, the mean age was 48,5 years (SD = 20,4) for the group of patients with infection without sepsis, and 57,2 years (SD = 20,2) for the septic shock group. In the first group, community-acquired infection accounted for 73,5% of cases, with infection of the urinary tract as the most frequent diagnosis in 31%, followed by soft-tissue infection with 29%; as opposite to the septic shock group, in which pneumonia was the most common infection with 29,4% of cases, followed by intraabdominal infection in 16.5% of patients (Table 1). More than half of the patients at all clinical stages had at least one comorbidity, with trauma or surgery as the most frequent. SOFA and APACHE scores, as well as the median length of hospital stay and 28-day mortality, increased proportionally with the progression of the clinical stages (Table 1). From day 1 to 2, the proportion of patients with infection without sepsis increased from 5% to 21% and the proportion of septic patients increased from 21% to 34%, whereas the proportion of patients with severe sepsis decreased from 59% to 32%. The frequency of septic shock did not change substantially, and maintained a similar distribution over the first week. Likewise, the proportion of discharges due to death reduced daily over the first week. It is noteworthy that most deaths during that period of time occurred at stages of severe sepsis and septic shock (Figure 1).

**Table 1:** General characteristics of study population according to initial clinical stage

**Characteristics**	**Infection without sepsis**	**Sepsis**	**Severe sepsis**	**Shock**
	**(n = 136)**	**(n = 575)**	**(n = 1576)**	**(n = 394)**
Age*	48.5 ± 20.4	49.3 ± 20.5	57.3 ± 20.1	57.2 ± 20.2
Male sex†	70 (51.5)	258 (44.9)	789 (50.1)	199 (50.5)
Community-acquired infection†	100 (73.5)	377 (65.6)	1120 (71.1)	249 (63.2)
Urinary tract infection†	42 (31.0)	120 (20.9)	369 (23.4)	40 (10)
Pneumonia†	9 (6.6)	101 (17.6)	417 (26.4)	116 (29.4)
Intra-abdominal infection†	4 (2.9)	27 (4.7)	161 (10.2)	65 (16.5)
Skin and soft tissues†	40 (29)	116 (20)	183 (11.6)	35 (8)
Trauma or surgery†	42 (30.9)	176 (30.6)	396 (25.1)	150 (38.1)
No comorbidities†	67 (49.3)	210 (36.5)	510 (32.4)	92 (23.4)
SOFA*	0.6 ± 0.8	1.2 ± 1.2	3.9 ± 2.4	8.0 ± 3.5
APACHE II*	4.4 ± 3.7	7.3 ± 4.9	12.4 ± 6.2	17.3 ± 6.7
Hospital stay‡	5 (2 – 39)	8 (1 – 77)	10 (1 – 79)	12 (1 – 74)
28-day mortality†	3 (2.2)	47 (8.2)	276 (17.6)	172 (43.6)

* Mean and standard deviation †Absolute frequency and percentage ‡Median and interquartile range.

### Risk of progression

The adjusted GEE model showed a 9% reduction of the odds of progression over the first week to more severe clinical stages in patients with skin and soft tissues infections, compared with those with urinary infection (OR = 0,91; 95% CI = 0,84-1,00); whereas intraabdominal infection increased the odds by 32% with regard to the same comparison group (OR = 1,32; 95% CI = 1,20-1,46). Other determinants of the odds of progression to more severe stages over the first week were the initial SOFA and APACHE II scores (OR = 1,16 [95% CI = 1,14-1,17] and OR 1,03 [95% CI = 1,02-1,03] for each point of increase, respectively), as well as respiratory infections and other sources of infection (Table 2). On univariate analysis, age (OR = 1.00; 95% CI = 1–1,01 by each year), male sex (OR = 1,07; 95% CI = 1-1,15), comorbidities (OR = 1,30; 95% CI = 1,21-1,40), and identification of Gram-negative microorganisms in blood (OR = 1,58; 95% CI = 1,41-1,75), among others, also seemed to increase the risk of progression, but such associations disappeared after adjustment for other covariables (Table 2).

**Table 2:** **Univariate and multivariate GEE models for estimating determinants of progression to sepsis, severe sepsis**, **or septic shock during the first week of hospital stay**

**Covariables**	**Univariate analysis**			**Multivariate analysis**		
	**OR**	**95% CI**	**P value**	**OR**	**95% CI**	**P value**
Age (for each year of increase)	1.00	(1.00 – 1.01)	<0.001	1.00	(1.00 – 1.00)	0.445
Sex						
Female	1	Reference		1	Reference	
Male	1.07	(1.00 – 1.15)	0.037	0.97	(0.92 – 1.02)	0.236
Site						
Community	1	Reference		1	Reference	
Hospital	1.12	(1.04 – 1.20)	0.002	1.03	(0.96 – 1.09)	0.409
Source of infection						
Urinary	1	Reference		1	Reference	
Respiratory	1.53	(1.38 – 1.68)	<0.001	1.21	(1.11 – 1.33)	<0.001
Skin and soft tissues	0.81	(0.73 – 0.91)	<0.001	0.91	(0.84 – 1.00)	0.041
Intra-abdominal	1.64	(1.46 – 1.84)	<0.001	1.32	(1.20 – 1.46)	<0.001
Other infections	1.33	(1.22 – 1.44)	<0.001	1.16	(1.08 – 1.25)	<0.001
Comorbidities						
Without comorbidities	1	Reference		1	Reference	
With any comorbidity	1.30	(1.21 – 1.40)	<0.001	1.01	(0.95 – 1.07)	0.774
SOFA score (0 – 24, for each unit of increase)	1.21	(1.20 – 1.22)	<0.001	1.16	(1.14 – 1.17)	<0.001
APACHE II score (0 – 67, for each unit of increase)	1.08	(1.07 – 1.08)	<0.001	1.02	(1.02 – 1.03)	<0.001
Blood cultures						
Not requested	1	Reference		1	Reference	
Negative	1.17	(1.09 – 1.27)	<0.001	0.96	(0.91 – 1.03)	0.281
Gram-positive bacteria	1.46	(1.28 – 1.66)	<0.001	0.99	(0.88 – 1.11)	0.867
Gram-negative bacteria	1.58	(1.41 – 1.75)	<0.001	1.01	(0.93 – 1.11)	0.748

### Risk of 7-day and 28-day mortality

The adjusted analysis of mortality during the first week with time-dependent covariates confirmed that the progression to severe sepsis and septic shock significantly increased the hazard of death: HR = 2,13; 95% CI = 1,13-4,03 and HR = 3,00; 95% CI = 1,5-5,98, respectively. Other independent determinants of mortality were age, respiratory source of infection, and initial SOFA and APACHE scores (Table 3). When verifying these findings with the outcome of 28-day mortality and clinical stages of infection as fixed variables at the moment of inclusion, only septic shock was significantly associated with mortality (HR = 2.10; 95% CI = 1,33-3,32). The risk of 28-day mortality was also associated with hospital-acquired infection, intraabdominal and other infection sources besides respiratory, age, and SOFA and APACHE scores (Table 4).

**Table 3:** Univariate and multivariate Cox models for 7-day mortality with clinical stage as time-dependent covariate

**Covariables**	**Univariate analysis**			**Multivariate analysis**		
	**HR**	**95% CI**	**P value**	**HR**	**95% CI**	**P value**
Age (for each year of increase)	1.02	1.01 – 1.02	<0.001	1.01	1.00 – 101	0.039
Sex						
Female	1	Reference		1	Reference	
Male	1.02	0.81 – 1.27	0.875	0.98	0.76 – 1.27	0.899
Site						
Community	1	Reference		1	Reference	
Hospital	1.29	1.02 – 1.62	0.034	1.33	0.98 – 1.80	0.064
Source of infection						
Urinary	1	Reference		1	Reference	
Respiratory	2.70	1.85 – 3.95	<0.001	1.76	1.12 – 2.77	0.014
Skin and soft tissues	1.07	0.68 – 1.69	0.776	1.22	0.73 – 2.02	0.441
Intra-abdominal	2.36	1.52 – 3.65	<0.001	1.50	0.90 – 2.48	0.118
Other infections	2.34	1.65 – 3.31	<0.001	1.89	1.28 – 2.80	0.001
Comorbidities						
Without comorbidities	1	Reference		1	Reference	
With any comorbidity	1.52	1.17 – 1.98	0.002	0.88	0.65 – 1.20	0.426
SOFA score (0 – 24, for each unit of increase)	1.24	1.21 – 1.27	<0.001	1.09	1.04 – 1.15	0.001
APACHE II score (0 – 67, for each unit of increase)	1.13	1.11 – 1.14	<0.001	1.07	1.04 – 1.10	<0.001
Blood cultures						
Not requested	1	Reference		1	Reference	
Negative	1.07	0.82 – 1.40	0.603	0.91	0.67 – 1.23	0.546
Gram-positive bacteria	1.37	0.88 – 2.11	0.158	0.83	0.50 – 1.36	0.454
Gram-negative bacteria	1.04	0.68 – 1.58	0.858	0.59	0.36 – 0.95	0.031
Classification of infection (progression to)						
Infection without sepsis	1	Reference		1	Reference	
Sepsis	2.75	1.47 – 5.15	0.002	1.76	0.93 – 3.33	0.081
Severe sepsis	4.95	2.71 – 9.06	<0.001	2.13	1.13 – 4.03	0.019
Septic shock	13.51	7.41 – 24.64	<0.001	3.00	1.50 – 5.98	0.002

**Table 4:** Univariate and multivariate Cox model for 28-day mortality with first-day clinical stage as fixed covariate

**Covariables**	**Univariate analysis**			**Multivariate analysis**		
	**HR**	**95% CI**	**P value**	**HR**	**95% CI**	**P value**
Age (for each year of increase)	1.02	1.01 – 1.02	<0.001	1.01	1.00 – 1.02	<0.001
Sex						
Female	1	Reference		1	Reference	
Male	1.07	0.90 – 1.28	0.446	1.02	0.84 – 1.23	0.829
Site						
Community	1	Reference		1	Reference	
Hospital	1.41	1.18 – 1.69	<0.001	1.38	1.10 – 1.72	0.005
Source of infection						
Urinary	1	Reference		1	Reference	
Respiratory	2.53	1.89 – 3.39	<0.001	1.75	1.25 – 2.45	0.001
Skin and soft tissues	1.13	0.80 – 1.59	0.483	1.30	0.91 – 1.87	0.151
Intra-abdominal	2.20	1.57 – 3.09	<0.001	1.60	1.10 – 2.32	0.013
Other infections	2.03	1.55 – 2.66	<0.001	1.72	1.29 – 2.30	<0.001
Comorbidities						
Without comorbidities	1	Reference		1	Reference	
With any comorbidity	1.64	1.33 – 2.03	<0.001	1.06	0.84 – 1.33	0.644
SOFA score (0 – 24, for each unit of increase)	1.20	1.18 – 1.23	<0.001	1.08	1.04 – 1.12	<0.001
APACHE II score (0 – 67, for each unit of increase)	1.11	1.09 – 1.12	<0.001	1.05	1.03 – 1.07	<0.001
Blood cultures						
Not requested	1	Reference		1	Reference	
Negative	1.22	0.99 – 1.50	0.058	1.06	0.84 – 1.32	0.623
Gram-positive bacteria	1.51	1.07 – 2.12	0.018	1.00	0.69 – 1.45	0.980
Gram-negative bacteria	1.55	1.16 – 2.07	0.003	1.02	0.75 – 1.40	0.894
Classification of infection (fixed at admission)						
Infection without sepsis	1	Reference		1	Reference	
Sepsis	2.08	1.40 – 3.09	<0.001	1.40	0.93 – 2.09	0.105
Severe sepsis	3.23	2.21 – 4.73	<0.001	1.50	0.99 – 2.26	0.052
Septic shock	7.79	5.31 – 11.42	<0.001	2.10	1.33 – 3.32	0.002

## Discussion

Our analysis showed that progression from infection to sepsis, severe sepsis or septic shock is strongly determined by the source of infection, independent of the initial values of SOFA and APACHE II scores. Similarly, this progression between clinical stages jointly with severity scores, age and the respiratory source of infection were the main determinants of mortality during the first week. We use specialized statistical models in order to fully explore the timeline of progression of the clinical stages of sepsis. Previously, Clermont et al. worked on dynamic microsimulation models to predict the temporal patterns of multiple outcomes in critically ill patients [23]. However, such approaches address mainly issues of prediction and forecasting and are not suitable enough for identification of individual covariates.

On the other hand, it has been reported in the literature that mortality has differential behavior according to the complexity and the source of the infection; with mortality rates usually higher at the stages of severe sepsis and septic shock, as well as in patients with pneumonia [1,3,5,8,24-27]. Our results confirm that septic shock as initial clinical stage, in addition to several sources of infection, increases the 28-day hazard of death. However, when stage shifts over the first week are assessed, it is clear that progression to severe sepsis and/or to septic shock are also independent determinants of mortality and the respiratory source of infection remained as a significant prognostic factor.

In order to compare our findings with those of other investigations, it is necessary to take into account all the differences of our study population. Our cohort consists of patients with community- and hospital-acquired infections, hospitalized in ICU or in general wards, whose mean age shows a young population, 33% of whose individuals have no comorbidities. In terms of the quantification of severity by SOFA and APACHE II scores, our data are comparatively low in comparison with those of other populations, even in the septic shock group [7-9,28-30].

Alberti et al. [8] published a multicenter study that enrolled 3443 ICU patients diagnosed with infection, 1531 of whom did not present with severe sepsis or shock. Eleven percent (n = 167) and 13% (n = 201) of these patients progressed to severe sepsis and septic shock, respectively, at some moment of their ICU stay before day 30. One of the chief factors of that progression to severe sepsis or septic shock was the source of infection; the risk was highest in cases of bacteremia (HR = 1,81; 95% CI =1,18-2,76), followed by peritonitis (HR = 1,51; 95% CI = 1,07-2,13) and pneumonia (HR = 1,47; 95% CI = 1,18-1,82). These results are in keeping with our own. Besides including only ICU patients and not estimating the effect of clinical progression on mortality, that study carried out an analysis based only on the time elapsed between admission and the appearance of severe sepsis or septic shock using a Cox model with competing risks. Such a model is not able enough to capture daily changes in clinical stages, which are outcomes that can appear repeatedly in a given patient during his/her hospital stay. Moreover, the authors found that growth of Gram-positive cocci and Gram-negative bacilli in blood increases the risk of progression to severe sepsis or septic shock during the first 30 days of ICU. In our study population, in which infections caused by Gram-negative bacilli were predominant in contradistinction to other current series across the world [11,31], the type of microorganism was not associated with progression to more advanced clinical stages. The apparent “protective association” of Gram-negative bacilli in blood with first-week mortality in our cohort is explained by a survival bias, given by a higher mortality in patients with Gram-negative bacteremia beginning during the second week. This was confirmed by the analysis with 28-day mortality, which showed no association with this type of microorganism (HR = 1,02; 95% CI = 0,75-1,40).

Dremsizov et al. [9] carried out a study in patients presenting to the emergency room with pneumonia, aimed at determining the onset and timing of severe sepsis and the ability of the Systemic Inflammatory Response Syndrome (SIRS) criteria and the Pneumonia Severity Index (PSI) to predict its development. Of 1339 patients with pneumonia, 882 did not have severe sepsis on admission, and 20,6% (n = 182) of these patients progressed to that stage at some moment of their hospital stay. The authors found that using 2 or 3 SIRS criteria does not predict the risk of progression to severe sepsis or septic shock or 30-day mortality, in contradistinction to PSI, which was associated with the development of severe sepsis. Besides considering only a specific type of infection and a specific type of risk score, that study did not analyze the factors from the perspective of their change over time, neither for exposure variables nor for outcome variables. Glickman et al. [32] carried out an investigation in patients presenting to the emergency room in order to determine the incidence, the mortality, and the factors associated with progression over the first 72 hours from sepsis to septic shock. The study included 472 patients without shock at the moment of evaluation in the emergency room, whose median age was 52 years and median APACHE II score was 9 points. The most common source of infection in the whole population was the respiratory, and the factors associated with progression to septic shock were catheter-related infection, age, female sex, temperature, and chronic lung disease. Besides the relative lack of ability of the logistic regression analysis to explore the behavior of variables that change over time, the study might have limitations related with the sample size necessary for a multivariable analysis.

The strengths of our study include the great diversity of clinical and epidemiologic aspects of its population, the quality of the data and the reproducibility of the definitions. Our study showed that severity scores not only increase with higher complexity of the infection [24-26] and predict mortality or organ dysfunction [13,14], which is what they were designed for, but also determine the risk of progression to more severe clinical stages. The latter suggest an additional advantage of severity scores, as potential tools for exploring the complexity of sepsis physiopathology. The limitations of our study include the lack of assessment of the impact of adequate treatment and resuscitation strategies on progression from one clinical stage to another [33,34], and the absence of biochemical markers used in clinical practice such as C-reactive protein, procalcitonin, or lactate.

## Conclusions

This study showed that source of infection, independent of the initial values of severity scores, increase the risk of progression to more severe stages of sepsis, and that there is a differential effect on first-week mortality according with those stages and their progression. Further studies are required to validate these conclusions and to identify interventions capable of modifying risk factors for progression as well as the mortality outcome in patients with severe infections that require management in hospital.

### Key messages

● The source of infection increase the risk of progression to more severe stages of sepsis.

● Intraabdominal and respiratory sources of infection, independently of SOFA and APACHE II scores, increase the risk of clinical progression to more severe stages of sepsis.

● There is a differential effect on first-week mortality according with those stages and their progression.

● Further studies are required to validate these conclusions.

## Competing interest

The authors declare that they have no conflict of interest.

## Authors’ contributions

ALLA, NAHV and FAJ conceived and designed the study, ALLA and FAJ provided statistical analysis, LIBV, GDRE, RDV, CDC, MGS, DLT, FART, FMJS and GOR recruited participants and supervised the study conduction at each hospital, ALLA and NAHV wrote the first draft of the manuscript, all the authors read and approve the last version of the paper. FAJ take responsibility for the investigation as a whole.

## Pre-publication history

The pre-publication history for this paper can be accessed here:

http://www.biomedcentral.com/1471-2334/13/345/prepub

## Supplementary Material

Additional file 1 Clinical definitions. Click here for file
