# Supplementary material for: Clinical course of sepsis, severe sepsis, and septic shock in a cohort of infected patients from ten Colombian hospitals
Source: BMC Infect Dis. 2013 Jul 24;13:345. doi: 10.1186/1471-2334-13-345 (PMC3727953; doi:10.1186/1471-2334-13-345)
Supplement: Additional file 1 — Clinical definitions. [file 1471-2334-13-345-S1.doc]

**Appendix 1.** Clinical definitions

**Infection**

Infection that fulfilled standard Centers for Disease Control and Prevention definitions

**Sepsis**

Infection that fulfilled standard Centers for Disease Control and Prevention definitions and one of the following:

A. Fever (core temperature ≥ 38.3°C) or hypothermia (core temperature ≤ 36°C)

B. Altered mental status (Glasgow Coma Scale <15)

C. Heart rate > 90 beats/min

D. Respiratory rate > 20 breaths/min

E. Significant edema or positive fluid balance (> 20 mL/kg over 24 hrs)

F. Plasma glucose > 120 mg/dL (7.7 mmol/L) in the absence of diabetes

G. White blood cell > 12,000 cells/µL (or > 15,000 cells/µL in postpartum women)

H. Plasma C-reactive protein ≥ 5 mg/dL

**Severe sepsis**

Is defined in the presence of the least one variable of organ dysfunction, hypoperfusion, or hypotension

*Organ dysfunction*

A. Arterial hypoxemia (PaO2/FIO2 < 300)

B. Acute oliguria (urine output <0.5 mL/kg/hr for at least 2 hrs)

C. Serum creatinine > 2 mg/dL

D. Coagulation abnormalities (international normalized ratio >1.5 or activated partial thromboplastin time > 60 secs)

E. Platelet count < 100,000 cells/µL

F. Plasma total bilirubin > 2 mg/dL

*Hypoperfusion*

A. Serum lactate > 2 mmol/L

B. Decreased capillary refill > 2 secs

C. Unexplained metabolic acidosis (arterial pH < 7.3)

*Hypotension*

A. Systolic blood pressure < 90 mmHg or a decrease < 40 mmHg with respect to previous values or mean arterial blood pressure < 60 mmHg

**Septic shock**

State of acute circulatory failure characterized by persistent arterial hypotension (as defined previously) despite adequate volume resuscitation or need of vasoactive drugs by continuous infusion over 6 hrs regardless of the values found in the initial measure of blood pressure

*Modified from Levy M, Fink M, Marshall J, Abraham E, Angus D, Cook D, et al. 2001 SCCM/ESICM/ACCP/ATS/SIS International Sepsis Definitions Conference. Critical Care Medicine. 2003;31(4):1250-6.*
